# Genome-Wide Characterization, Evolution, and Expression Profile Analysis of GATA Transcription Factors in *Brachypodium distachyon*

**DOI:** 10.3390/ijms22042026

**Published:** 2021-02-18

**Authors:** Weiye Peng, Wei Li, Na Song, Zejun Tang, Jing Liu, Yunsheng Wang, Sujun Pan, Liangying Dai, Bing Wang

**Affiliations:** 1 Hunan Provincial Key Laboratory for Biology and Control of Plant Diseases and Insect Pests, Hunan Agricultural University, Changsha 410128, China; 15580076854@163.com (W.P.); liwei350551@163.com (W.L.); chinasong86@126.com (N.S.); Tangzj516@126.com (Z.T.); liujing3878@sina.com (J.L.); wyunsheng@gmail.com (Y.W.); sujunpan@126.com (S.P.); 2 College of Plant Protection, Hunan Agricultural University, Changsha 410128, China

**Keywords:** *Brachypodium distachyon*, GATA transcription factors, genome-wide characterization, phylogenetic analysis, profile analysis

## Abstract

The GATA proteins, functioning as transcription factors (TFs), are involved in multiple plant physiological and biochemical processes. In this study, 28 GATA TFs of *Brachypodium distachyon* (BdGATA) were systematically characterized via whole-genome analysis. *BdGATA* genes unevenly distribute on five chromosomes of *B. distachyon* and undergo purifying selection during the evolution process. The putative *cis*-acting regulatory elements and gene interaction network of BdGATA were found to be associated with hormones and defense responses. Noticeably, the expression profiles measured by quantitative real-time PCR indicated that *BdGATA* genes were sensitive to methyl jasmonate (MeJA) and salicylic acid (SA) treatment, and 10 of them responded to invasion of the fungal pathogen *Magnaporthe oryzae*, which causes rice blast disease. Genome-wide characterization, evolution, and expression profile analysis of *BdGATA* genes can open new avenues for uncovering the functions of the *GATA* genes family in plants and further improve the knowledge of cellular signaling in plant defense.

## 1. Introduction

GATA transcription factors (TFs), as important regulatory proteins for gene expression and biological processes, are widely distributed in eukaryotes, including animals, plants, and fungi [1]. GATA family TFs are well characterized as specifically recognizing a conserved DNA motif (T/A)GATA(G/A), and their DNA binding domain has one or two typical Cys2/Cys2 type-IVb zinc finger motifs [2]. *NtGATA1*, homologous to the *Neurospora crassa nit-2* gene, was the first identified and cloned member of the GATA TFs found in tobacco [3]. As a subsequent study, GATA TFs were determined and classified into four subfamilies (A to D) on the basis of the evolutionary relationship and structure domains [4]. There are distinctions among the zinc loop motifs found in the four GATA subfamilies: subfamily A contains a CX_2_CX_18_CX_2_C zinc loop; subfamily B possesses a similar zinc loop, except for the four amino acid motifs (XTNMMX) existing between the first and second Cys; subfamily C contains a CX_2_CX_20_CX_2_C and a TIFY motif related to biological clock and hormone signaling responses; and subfamily D possesses a CX_2_CX_18_CX_2_C zinc loop at the N-terminal end. In addition, the exon numbers of these GATA TFs subfamilies are distinct, with two exons in subfamily A, three in subfamily B, seven in subfamily C, and six in subfamily D [5].

GATA TFs are reported to be widely involved in regulating the processes of plant growth and secondary metabolism. *PdGATA19* in *Populus* plays multiple crucial roles in secondary xylem differentiation and plant growth, predicted by *cis*-acting regulatory elements (CREs) [6]. Overexpression of *PdGATA19* in *Populus* plants showed added biomass accumulation, increased stem height and photosynthetic rate, and 20% higher chlorophyll content; on the other hand, CRISPR/Cas9-mediated mutant *pdgata19* exhibited severe growth retardation and a higher proportion of secondary xylem [6]. There is a similar situation with rice and *Arabidopsis*—GATA TFs are related to chlorophyll content and plant growth, and even yield [7,8]. For *Arabidopsis* GATA12 as a target of RGA-LIKE2 (RGL2, a pivotal transcriptional repressor of the gibberellic acid signaling pathway), its transcription level was negatively regulated by gibberellic acid (GA) and sharply reduced in specific broken dormancy environments of cold stratification and dry storage of seeds [9,10]. In addition, the seeds derived from *GATA12* suppressed transgenic plants exhibiting a lower degree of dormancy, while seeds from overexpressed *GATA12* lines showed a high degree of dormancy [10].

Plants frequently encounter various hormones and stresses in their ecosystems. Jasmonate ZIM-domain (JAZ) protein, a key regulator of the jasmonate acid signaling pathway, is capable of interacting with GATA TFs [11]. The GATA TF *LESION SIMULATING DISEASE1* (*LSD1*) was originally discovered in *Arabidopsis*, which is related to the salicylic acid (SA) signaling pathway and negatively regulates programmed cell death; the expression level of *LSD1* affects the expression of SA signaling-related genes [12]. It is reported that GATA TFs play a significant role in resistance to abiotic stress [13,14,15]. For example, the expression of the Arabidopsis GATA TF gene GATA nitrate-inducible, carbon metabolism-involved (AtGNC) in low-concentration nitrogen treatment is about 1.5 folds higher than that in high-concentration nitrogen treatment [16]. Similarly, overexpressed lines of the rice GATA TF gene CYTOKININ-RESPONSIVE GATA FACTOR1 (*OsCga1*) under a nitrogen-deficient environment are still able to sustain increased chlorophyll content, but not the *oscga1* mutant [17]. Moreover, *GATA* genes play an important role in biotic stress. It is reported that the GATA proteins are involved in the rice–*Magnaporthe oryzae* interaction, validated by gene expression profiling [18]. An *Arabidopsis* GATA family protein, At4G20380, participates in the plant hypersensitive response (HR) to pathogen invasion [19]. Additionally, TF clique analysis indicated that GATA TFs co-occurred with WRKY and NAC TFs to regulate plant immunity [20].

*Brachypodium distachyon* is an ideal model monocot plant for studying interactions with fungi, which can be infected by multiple cereal pathogens, such as *M. oryzae*. In this study, we performed a genome-wide survey of *GATA* genes in *B. distachyon* and conducted an expression profiling analysis of all *GATA* genes with hormone treatments and *M. oryzae* inoculation. It was found that 10 GATA genes in *B. distachyon* responded to fungal infection. The findings obtained in this study elucidate the evolutionary process of GATA proteins in *B. distachyon*, which will provide evidence for further study of *GATA* gene functions.

## 2. Results

### 2.1. Identification and Phylogenetic Analysis of GATA Family Proteins in Brachypodium Distachyon

In this study, a total of 28 proteins with complete GATA domain were found in *B. distachyon*. The open reading frame (ORF) length of *B. distachyon* GATA TFs varied from 252 to 1635 bp, and the theoretical molecular weight of the putative GATA proteins ranged from 9 to 59 kDa (Supplementary Table S1).

**Figure 1:**
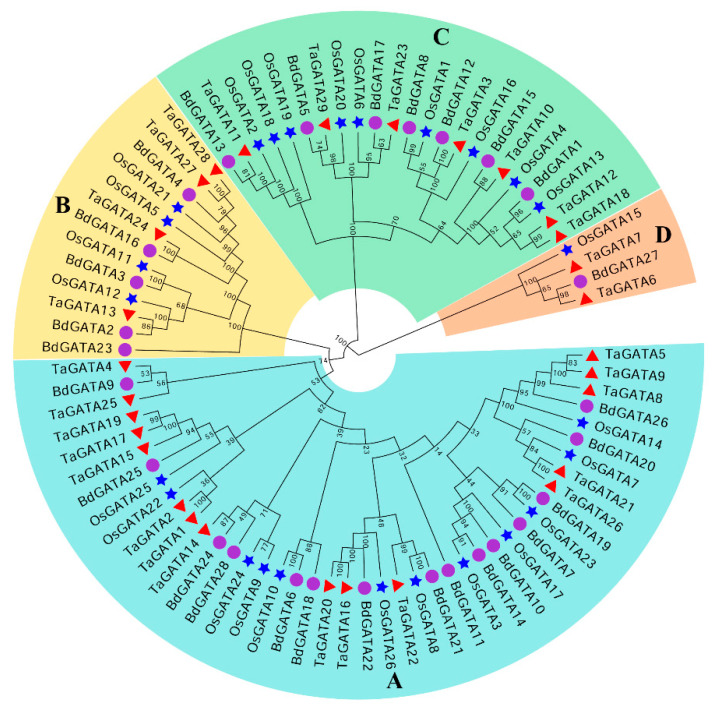
Phylogenetic tree of GATA proteins from *Brachypodium distachyon*, rice, and wheat. Phylogenetic relationships of GATA proteins from *Brachypodium distachyon* (28), rice (26), and wheat (29) were performed with MEGA v5.0 using the NJ method with 1000 bootstrap replicates. The GATA proteins were clustered into four major groups—A, B, C, and D. Members of *B. distachyon*, rice, and wheat are represented by purple circles, blue stars, and red triangles, respectively. Gene IDs of GATAs from *B. distachyon*, rice and wheat were listed in Table S2.

In order to analyze the evolutionary diversity of *GATA* genes, we constructed a comprehensive neighbor-joining phylogenetic tree for the three Gramineae species *B. distachyon*, rice (*Oryza sativa* L.), and wheat (*Triticum aestivum* L.), and the locus names of the *GATA* genes from these three are listed in Supplementary Table S2. The phylogenetic clustering analysis revealed that *GATA* genes were divided into four subgroups, named A to D according to their motifs (Figure 1). Among them, group A accounted for over half of the total (53.5%) and group D for only one member (*BdGATA27*) of *B. distachyon*. Group B contained the integrated conserved domain (GATA/CCT/TIFY); however, group D lacked some motifs. Consistently, the vast majority of GATA proteins within the same group had similar physicochemical characteristics, as shown in Table S1.

### 2.2. Chromosome Location and Duplication Analysis of BdGATA Genes

**Figure 2:**
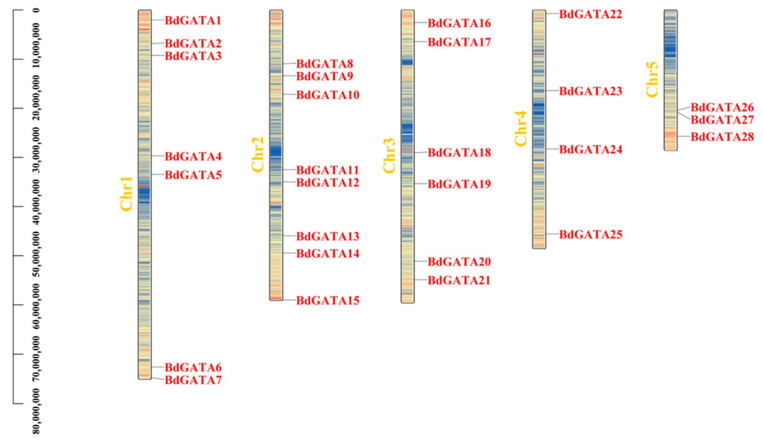
Chromosomal locations of *BdGATA* with gene density. The size of a chromosome is estimated from its relative length. The chromosome numbers are shown on the left of each chromosome. The gene density is indicated by the color bar (blue-to-red scale indicates gene density from low to high).

**Figure 3:**
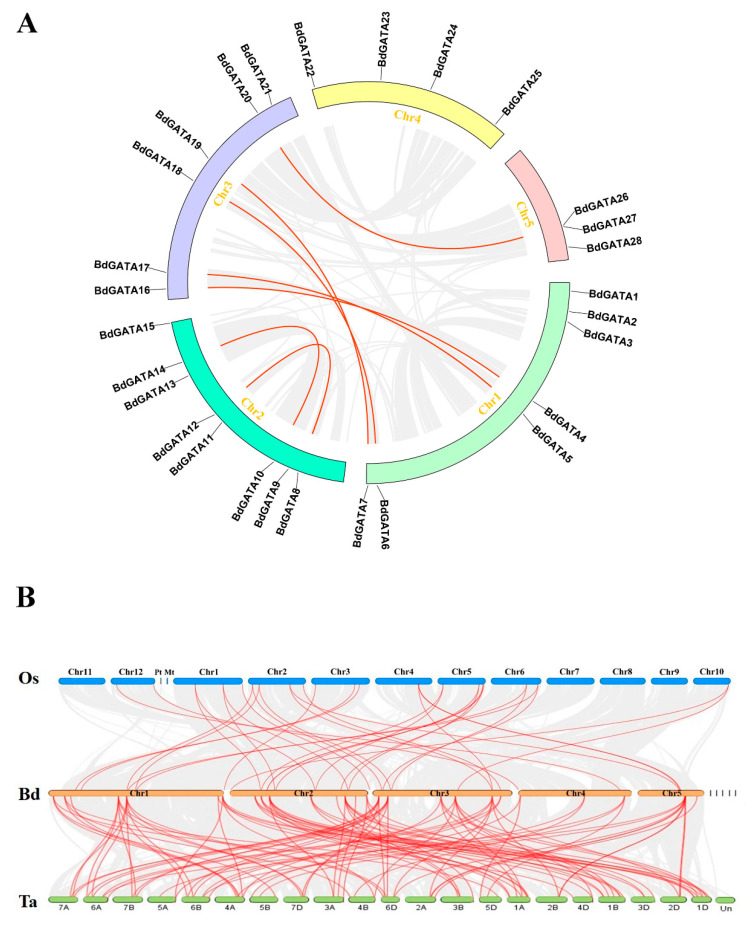
Duplication pairs and synteny analysis of *GATA* genes between *Brachypodium distachyon* and rice/wheat. (**A**) Each chromosome is represented with a different color. Gray curves denote the details of syntenic regions in *Brachypodium distachyon* genome and red curves denote *BdGATA* gene pairs with segmental duplication. (**B**) Red lines indicate homologous genes between *Brachypodium distachyon* and rice/wheat chromosomes.

To further determine the chromosome position distribution of *BdGATA* genes, we utilized the tblastn program of the National Center for Biotechnology Information database to search for the nucleotide sequence of each *BdGATA* gene. The distribution of blast hits is shown in Figure 2. The *GATA* genes were non-uniformly distributed in the five chromosomes of *B. distachyon*, and there was no biological relevance between *BdGATA* gene number/density and the chromosome, the maximum of which is eight *BdGATA* genes existing on chromosome 2, with a minimum of three on chromosome 5 (Figure 2). For the vast majority of species, tandem and segmental duplication are the crucial evolutionary driving forces [21]. Therefore, we analyzed the tandem and segmental duplication characteristics of *GATA* genes and identified a *BdGATA* tandem duplication cluster (*BdGATA26/BdGATA27*). Moreover, seven *BdGATA* gene pairs were found as segmental duplication events (Figure 3A, Supplementary Table S3), which may partially contribute to *GATA* gene generation. The rate of nonsynonymous (Ka) to synonymous (Ks) substitution was used to evaluate the tendency and strength of natural selection action. The Ka/Ks values of all *GATA* gene pairs were less than 1.00 (0.28–0.90), which indicates that the *GATA* gene pairs in *B. distachyon* undergo intense purifying selection during the evolutionary process (Supplementary Table S3). Moreover, we also calculated the approximate date of duplication events. The duplication events of *BdGATA* genes occurred from 53.88 Mya (Ks = 0.700) to 89.75 Mya (Ks = 1.166), with a mean of 73.53 Mya (Ks = 0.955). To deduce the synteny of *GATA* genes, we conducted a collinearity analysis between *B. distachyon* and the other two cereal species (rice and wheat) using One Step MCScan. Consequently, 33 paired collinearity relationships between *BdGATA* (*B. distachyon*) and *OsGATA* (rice) genes were found, and there were 98 pairs between *BdGATA* and *TaGATA* (wheat) genes (Figure 3B). Detailed information of the synteny analysis, including gene pairs and chromosome locations, is shown in Supplementary Table S4.

### 2.3. Structures and Conserved Motifs of BdGATA Genes

**Figure 4:**
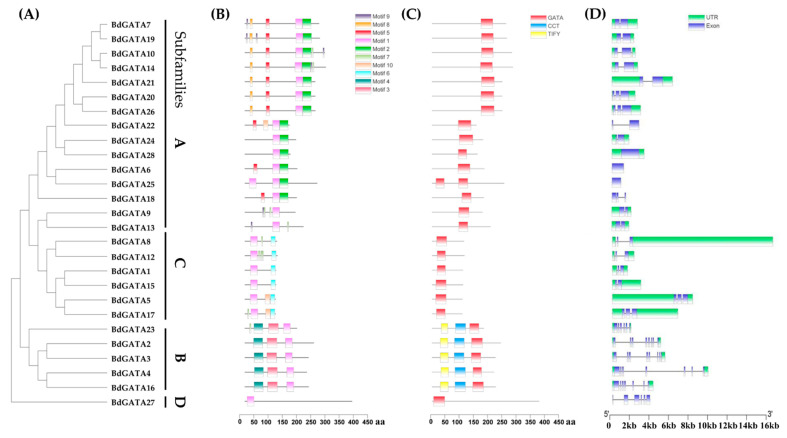
Putative conserved motifs, domains, and gene structures of the *BdGATA* family. (**A**) Phylogenetic tree. Multiple sequence alignment of BdGATA proteins was performed using ClustalX. The neighbor-joining tree was constructed using MEGA v5.0 with 1000 bootstrap replicates. (**B**) Conserved motifs. MEME Suite version 5.1.1 revealed the conserved motifs of the GATA proteins. (**C**) The distribution of conserved domains of BdGATA family. aa: amino acids. (**D**) Gene structure. The blue square, green square, and black lines represent the exon, untranslated region (UTR), and intron, respectively.

To illustrate the potential correlation between gene structure–function and the evolution process of *BdGATA* genes, we analyzed gene structure (exons/introns) and conserved domains/motifs. As shown in Figure 4, we found that the structures of *GATA* genes in the same subcluster were similar, which can also serve as additional evidence to support the classification of *BdGATA* genes. In the BdGATA proteins, 10 motifs were identified and defined as motifs 1 to 10 (Figure 4B and Supplementary Figure S1). In addition, besides the common GATA domain, there were two additional domains, CCT and TIFY, exclusively present in subfamily B (Figure 4C). In detail, *BdGATA2/3/4/16/23* contains similar structures and multiple exons, usually six to seven, whereas *BdGATA24*/*25*/*28* contains only one exon (Figure 4D). This, again, reveals the occurrence of duplication events. On the other hand, significant differences in the number of exons indicate gain or loss of DNA fragments throughout the evolutionary process.

### 2.4. Prediction of CRE-Involved Pathways, BdGATA Targets of miRNA, and BdGATA Interaction Network

To illuminate the mechanisms of *GATA* genes in transcriptional regulation, we identified the CREs within a region 2 kb upstream from the start codon (ATG) of *GATA* genes using the PlantCARE website search tool. The predictions showed that the CREs of *BdGATA* genes are mainly involved in 11 biological pathways and that each *GATA* gene contains several types of CREs (Supplementary Figure S2, Supplementary Table S5) that participate in responses to stress.

**Figure 5:**
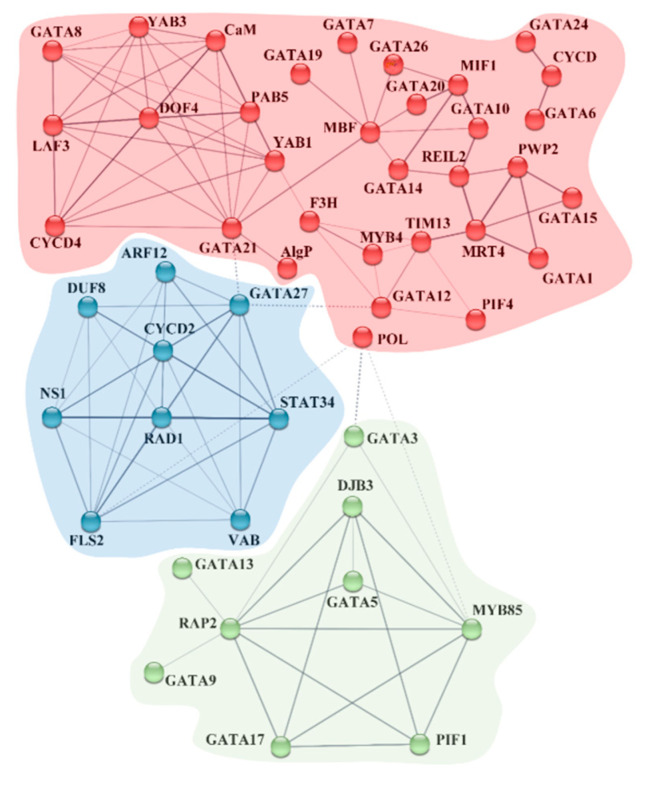
Interaction network of GATA and related genes. Line thickness indicates the strength of data support. The associations were inferred from the evidence from the STRING database—known interactions, predicted interactions, and others.

To further reveal the potential interactions between BdGATA proteins and other proteins, we drafted the BdGATA protein interaction network on the basis of the String database (Figure 5, Supplementary Table S6). The network is clustered into three sets, which were divided according to K-means. Several BdGATA proteins directly or indirectly interact with each other (e.g., BdGATA12–BdGATA21–BdGATA27). Coincidently, both related to AP2-like ethylene-responsive transcription factor 2 (RAP2) and multiprotein bridging factor (MBF) were predicted to interact with five BdGATA proteins in varying degrees.

Numerous microRNAs (miRNAs) have a strong tendency to target genes involved in defense and development, especially TF genes [22]. A previous report demonstrated that some GATA genes were potential regulatory targets of miRNAs [23]. To rapidly predict *GATA* genes that are possible targets of miRNA, we performed a web-based plant small RNA target analysis (Supplementary Table S7). The results revealed that 16 *BdGATA* genes were predicted targets of 13 miRNAs. It should be noted that potato miR2673 targets seven *GATA* genes, and miR2673-GATA (PGSC id: DMT400024205) in potato was predicted to regulate resistance against late blight disease [24]. Furthermore, miR2673 was reported to implicate plant oxidative burst and HR via proline dehydrogenase [25].

### 2.5. Expression Profile of BdGATA Genes in Response to Magnaporthe Oryzae and Hormone Treatments

**Figure 6:**
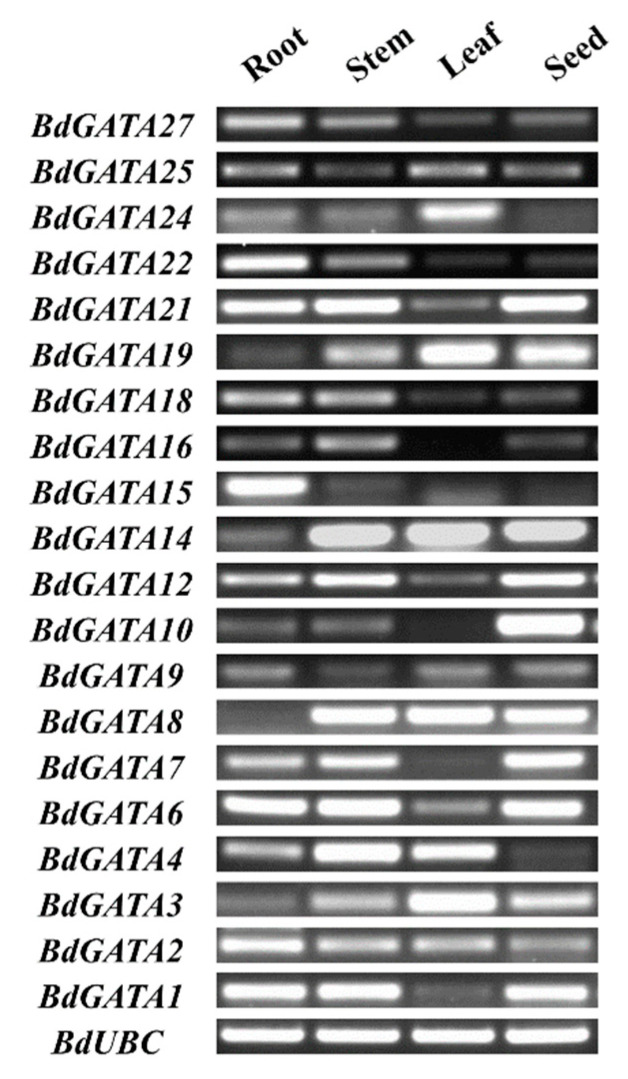
RT-PCR analysis of *BdGATA* expression in different tissues of *Brachypodium distachyon.*

To detect the *BdGATA* gene expression profile in specific *B. distachyon* organs (roots, stems, leaves, and seeds), we performed a reverse-transcription polymerase chain reaction (RT-PCR) with the *BdUBC* gene as the internal control. As shown in Figure 6, *BdGATA* genes had different expressions in leaves, roots, stems, and seeds. For example, *BdGATA1/6/21* showed similar expression patterns, which were at a high level in roots, stems, and seeds, but a low level in leaves. *BdGATA2/25/27* were ubiquitously expressed in all tested organs, with varying transcript levels.

**Figure 7:**
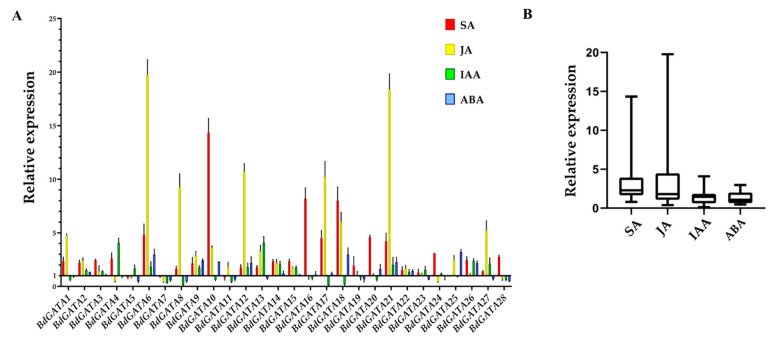
Expression profiles of *BdGATA* genes with phytohormone treatments. (**A**) Fold change in expression of BdGATA genes after hormone treatments. Fold change in BdGATA gene expression after hormone treatments assessed by qRT-PCR of cDNA isolated from hormone treatment plants were compared with mock treatment plants. Fold change of 1 means no difference in gene expression between hormone treatment plants and mock treatment plants. This assay was repeated three times with similar results. (**B**) Box–whisker plot of *BdGATA* gene expression after phytohormone treatments. The *y*-axis represents relative gene expression level. Whiskers represent maximum/minimum values, boxes indicate the first quartile and third quartile, and the horizontal line represents the median.

The expression of *BdGATA* genes was detected after treatment with different phytohormones. Compared to the other three hormone treatments, *BdGATA10/16/18/20/24/28* showed more significant expression change after SA treatment. In addition, *BdGATA6/8/12/17/21/27* showed significant expression change upon JA treatment (Figure 7A). To make the results more intuitive, we have shown the results of *BdGATA* gene expression after phytohormone treatments as a box–whisker plot containing a median (line) and interquartile range (box ends). According to Figure 7B, one can see clearly that *BdGATA* genes were strongly induced by JA and SA, but not abscisic acid (ABA) and indoleacetic acid (IAA).

**Figure 8:**
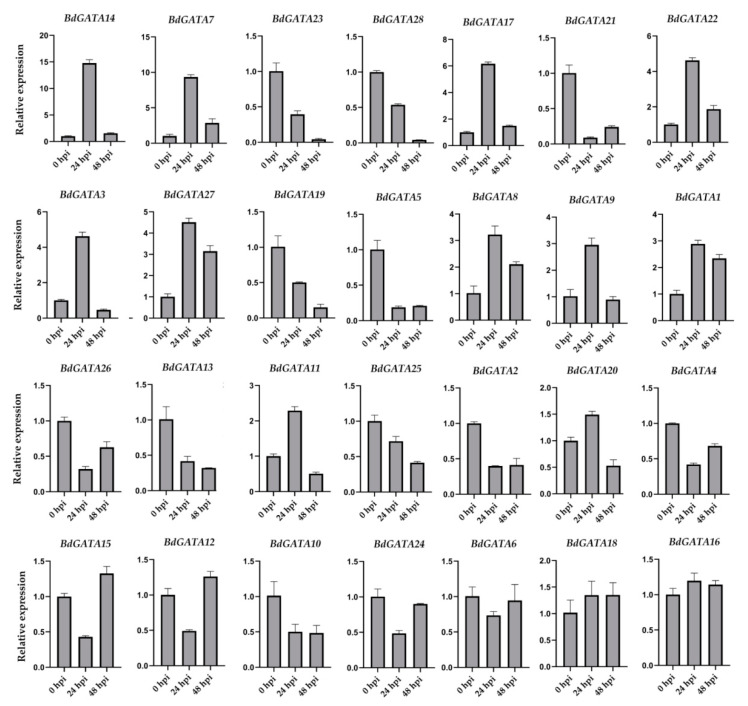
Expression of *BdGATA* genes inoculated with *Magnaporthe oryzae*. The relative expression levels of *BdGATA* genes were normalized with *BdUBC*. The experiments in all panels were repeated three times with similar results.

In addition, the transcription levels of *BdGATA* genes were detected in *B. distachyon* inoculated with *M. oryzae* isolate RO1-1 at 0, 24, and 48 h post inoculation (hpi). The relative expression of some *BdGATA* genes displayed significant induction at 24 and/or 48 hpi, such as *BdGATA1/7/8/9/11/14/17/22/27*; however, several *GATA* genes were induced at 24 but not 48 hpi (Figure 8). On the contrary, some *BdGATA* genes were suppressed after inoculation, for instance, *BdGATA2/4/5/10/13/19/21/25/26/28* (Figure 8). On the whole, most of the BdGATA proteins might participate in *B. distachyon* disease resistance responses.

## 3. Discussion

The GATA TFs participate in the regulation of various vital physiological and biochemical processes in plants [26]. However, the functions and mechanisms of GATA TFs in regulating hormone pathways and plant immunity are still largely unknown. In this study, we conducted a genome-wide analysis of the *GATA* gene family to explore their potential functions and expression diversity upon hormone and pathogen infection in *B. distachyon*. A total of 28 *GATA* genes were presumed throughout the whole genome of *B. distachyon*, which is close to the 31 GATA proteins identified in *Malus domestica* [27] and 28 GATA proteins identified in rice [28]. According to phylogenetic analysis, we classified *GATA* genes identified from *B. distachyon* into four subfamilies, which is consistent with classification in other plant species, such as the *Gossypium* genus and *M. domestica* [29]. *BdGATA* genes are widely expressed in different organs of *B. distachyon*, which suggests the important roles of BdGATA in plants. *BdGATA11* is a pseudogene, considering it has large deletions and no detectable expression in any organs.

To explore the evolutionary pattern of GATA, we calculated selective pressure. The values of the Ka/Ks ratio for all BdGATA duplicated pairs were less than 1, indicating that purifying selection played a significant role in the evolution of *BdGATA* genes. In terms of multiple sequences alignment, subfamily B of *BdGATA* genes contain two additional domains, CCT and TIFY, in addition to the fully conserved GATA domain. This is consistent with the conclusion previously reported for dicot plant *Brassica napus* and monocot plant *Zea mays* [30,31].

Hormones are essential signaling molecules that regulate plant immune responses and development [32]. In this study, *BdGATA* genes showed diverse expression patterns after SA, JA, ABA, and IAA treatment. We suggest that an analysis of *GATA* genes expression profile under hormone treatment could provide an effective reference for the following screening genes related to rice blast resistance.

*B. distachyon* is host to a range of cereal pathogens. For example, the *Brachypodium–M. oryzae* interaction closely models the rice*–M. oryzae* interaction [33,34]. In the prediction of BdGATA interacting proteins, RAP2 and MBF proteins exhibited strong associations with GATA proteins. MBF functions as a key regulator of resistance to *Pseudomonas syringae* pv. *tomato* DC3000 and *Puccinia striiformis* f. sp. *tritici* by activating pathogenesis-related genes, SA, and ethylene pathways [35,36]. Likewise, RAP2 was reported to be an active transcriptional repressor of elevated *Botrytis cinerea* resistance and responses to ethylene [37]. All of these data suggest that BdGATA is likely to regulate disease resistance together with the interacting genes. In this study, on the basis of expression patterns in response to hormone treatment and *M. oryzae* inoculation, we found that several *B. distachyon GATA* genes were upregulated upon both treatments, including *BdGATA1/8/14/19*, which suggests that these upregulated *GATA* genes could contribute to regulating plant disease resistance against *M. oryzae*.

In addition, the transcription levels of duplicated *BdGATA* gene pairs exhibited similar expression patterns, indicating that their role could be redundant. The functional redundancy of duplicated genes may be associated with the analogous CREs, gene models, and conserved motifs [38]. For instance, BdGATA7/19 duplicated pairs showed similar tertiary structures and expression patterns in response to *M. oryzae* inoculation, and both are predicted to interact with MBF proteins. At any rate, further study of the functions and molecular mechanisms of *BdGATA* on plant disease resistance and hormone signaling transduction would be valuable.

## 4. Materials and Methods

### 4.1. Plant Materials, Hormone Treatments, and Rice Blast Inoculation

*B. distachyon* Bd21-3 genotype seeds were germinated on wet filter paper in a culture dish for 3 days and planted into a float tray containing vermiculite-based soil in a 22 °C greenhouse [39]. Seedlings were supplied with nutrient solution weekly for 2 months until processing.

For hormone treatment, 30-day-old *B. distachyon* plants were sprayed with 100 µM jasmonic acid (JA), 100 µM salicylic acid (SA), 100 µM indoleacetic acid (IAA), 100 µM abscisic acid (ABA), or distilled H_2_O as mock treatment, and then the leaves were sampled 2 h after treatment [40].

### 4.2. Rice Blast Inoculation

*M. oryzae* isolate RO1-1 was grown on oat medium and incubated at 26 °C for about 15 days until spores germinated. Subsequently, 30-day-old *B. distachyon* plants were inoculated with 1 × 10^6^ spores/mL using a spraying method as previously described [41]. Samples were harvested at 0, 24, and 48 h post-inoculation and kept at −80 °C until subsequent RNA extraction.

### 4.3. Identification of Brachypodium Distachyon GATA Gene Family and Phylogenetic Analysis

Information and sequences of *B. distachyon* GATA family genes were obtained from the Plant Transcription Factor Database v5.0 (http://planttfdb.cbi.pku.edu.cn/family). Annotation files in general feature format (.gff) and gene transfer format (.gtf) were retrieved from EnsemblPlants (https://plants.ensembl.org/info/website/ftp/index.html). The genome sequence was accessed from https://www.ncbi.nlm.nih.gov/genome.

Multiple alignments of amino acid sequences were conducted using the ClustalX program (version 2.08) with default parameters. A phylogenetic tree was generated with MEGA v5.0 software by the neighbor-joining (NJ) method, including pairwise deletion, Poisson distribution, and 1000 bootstrap replicates.

### 4.4. Gene Structure, Synteny Analysis, and Bioinformatics Resources

The theoretical isoelectric point (pI) and molecular weight (MW) of the deduced protein were calculated with the ExPASy web tool (https://web.expasy.org/compute_pi/). Motifs were identified by web-based MEME Suite v5.1.0 software (https://meme-suite.org/meme/) with standard parameters. Protein structural domain data were detected from the online conserved domain (CD) search tool (https://www.ncbi.nlm.nih.gov/Structure/bwrpsb/bwrpsb.cgi), with an expected value of  ≤0.01.

The chromosomal locations of the TF genes were learned from TBtools v0.6683 (a program for annotated gene locations from GFF3/GTF files). The multiple alignments were performed using the procedure of the One Step MCScanX with genome sequence file (.fa) and genome structure annotation file (.gff); subsequently, the collinearity results were depicted by the dual synteny plotter for MCScanX program, and two steps above were realized by Toolkit for Biologists (github.com/CJ-Chen) [42].

For *cis*-acting regulatory element analysis, we extracted 2000 bp DNA sequences upstream from the translation initiation site and then analyzed these regions in PlantCare (http://bioinformatics.psb.ugent.be/plantcare/html) [43]. We performed miRNA target analysis using the psRNATarget web tool (http://plantgrn.noble.org/psRNATarget) [44]. For retrieval of interacting proteins, protein sequences were analyzed using the STRING web portal (http://string-db.org) [45].

### 4.5. RNA Extraction and qRT-PCR Analysis

Total RNA of *B. distachyon* was extracted using TRIzol Reagent (Ambion, Waltham, MA, USA), and purified with an *EasyPure* Plant RNA Kit (TianGen, Beijing, China). Subsequently, cDNA first-strand synthesis was conducted by reverse transcription using a NovoScript Plus All-in-one SuperMix kit (Novoprotein, Shanghai, China). Specific primers for RT-PCR and qRT-PCR are listed in Supplementary Table S8. The roots, stems, leaves, and seeds were sampled at the same time intervals. To investigate the expression of *BdGATA* genes in different tissues/organs, we carried out the method for tissue sample collection as previously described [46]. RT-PCR was conducted using a MonScript RTase II kit (Monad, Suzhou, China). PCR was performed with 10–15 pmol of each primer, DNA templates (60 ng), double-distilled H_2_O, and Hieff PCR Master Mix (Yeasen, Shanghai, China) in a total volume of 20 μL. qRT-PCR was performed using a TransStart Tip Green qPCR SuperMix kit (TransGen, Beijing, China) in a CFX 96 qPCR instrument (BioRad, Hercules, CA, USA). *BdUBC* (*Bradi4g00660*) was amplified as the normalization control.

## 5. Conclusions

Genome-wide characterization of the *GATA* gene family in *B. distachyon* was performed, with special emphasis on the response to rice blast infection. A total of 28 *B. distachyon GATA* genes were identified and grouped into four subfamilies. The chromosomal location, gene duplication, structural features, conserved domain and motif, CREs, miRNA target, and expression profiles (organ-specific, response to *M. oryzae* and hormone treatment) of the *GATA* gene were analyzed. In conclusion, the 28 predicted *BdGATA* genes were classified into four subfamilies in this study, on the basis of their motifs. The *BdGATA* genes, widely expressed in different organs, were responsive to SA and JA hormone signaling pathways, and 10 of them responded to invasion of the fungal pathogen *M. oryzae*, which indicates that they are involved in plant immunity. In addition, the purifying selection played a significant role in the evolution of *BdGATA* genes. Overall, these investigations and findings provide a foundation for further study on the disease resistance functions of the GATA family and present new ideas for the breeding of disease-resistant plants.

## Data Availability

Data is contained within the article or supplementary material.

## Supplementary Materials

The following are available online at https://www.mdpi.com/1422-0067/22/4/2026/s1.
