# Supplementary figures and images for: Genome-Wide Characterization, Evolution, and Expression Profile Analysis of GATA Transcription Factors in Brachypodium distachyon
Source: Int J Mol Sci. 2021 Feb 18;22(4):2026. doi: 10.3390/ijms22042026 (PMC7922913; doi:10.3390/ijms22042026)

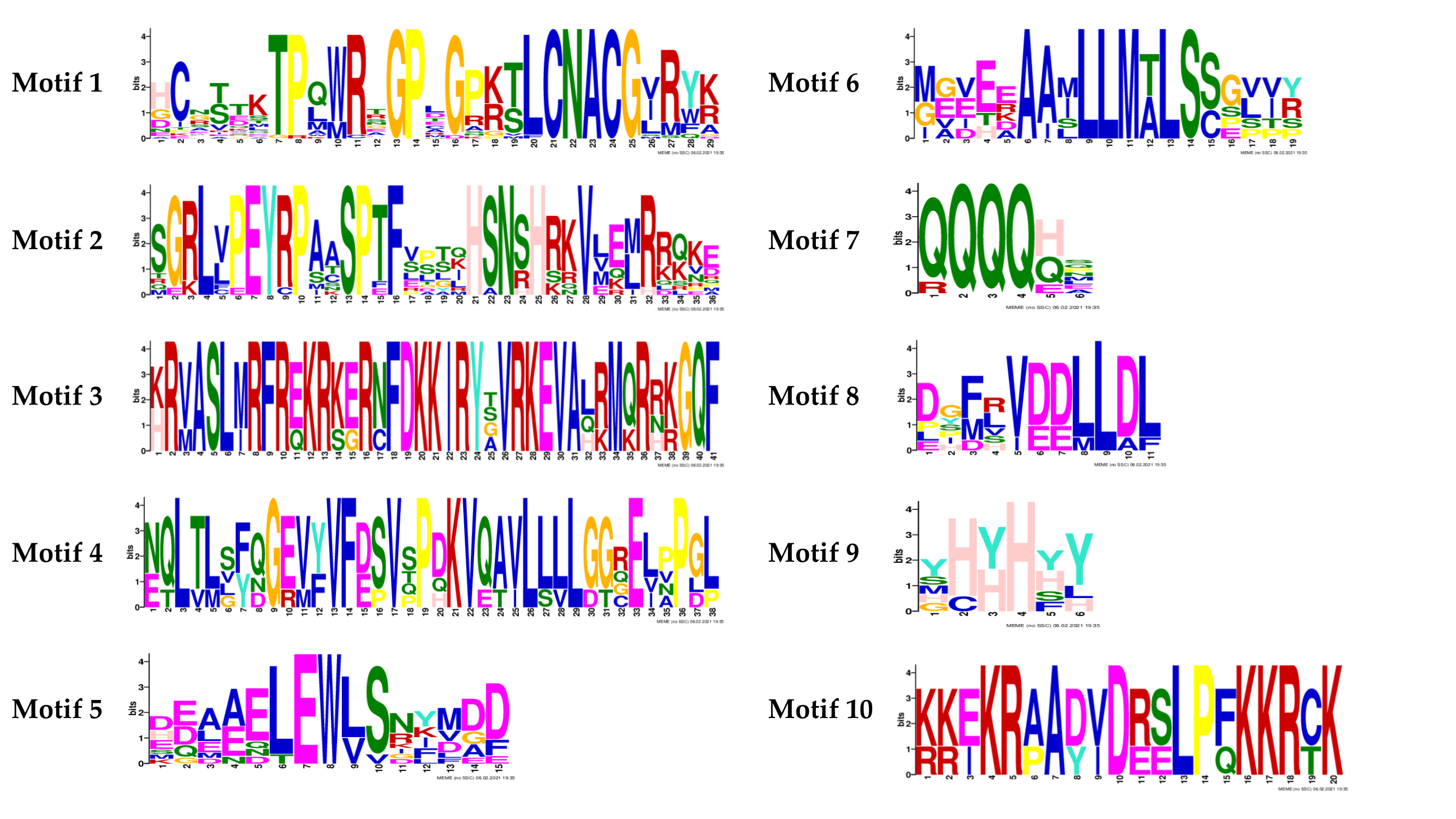

Supplement: Supplementary file 1 [file ijms-22-02026-s001.zip › supps resubmitted 2/Figure S1.tiff]

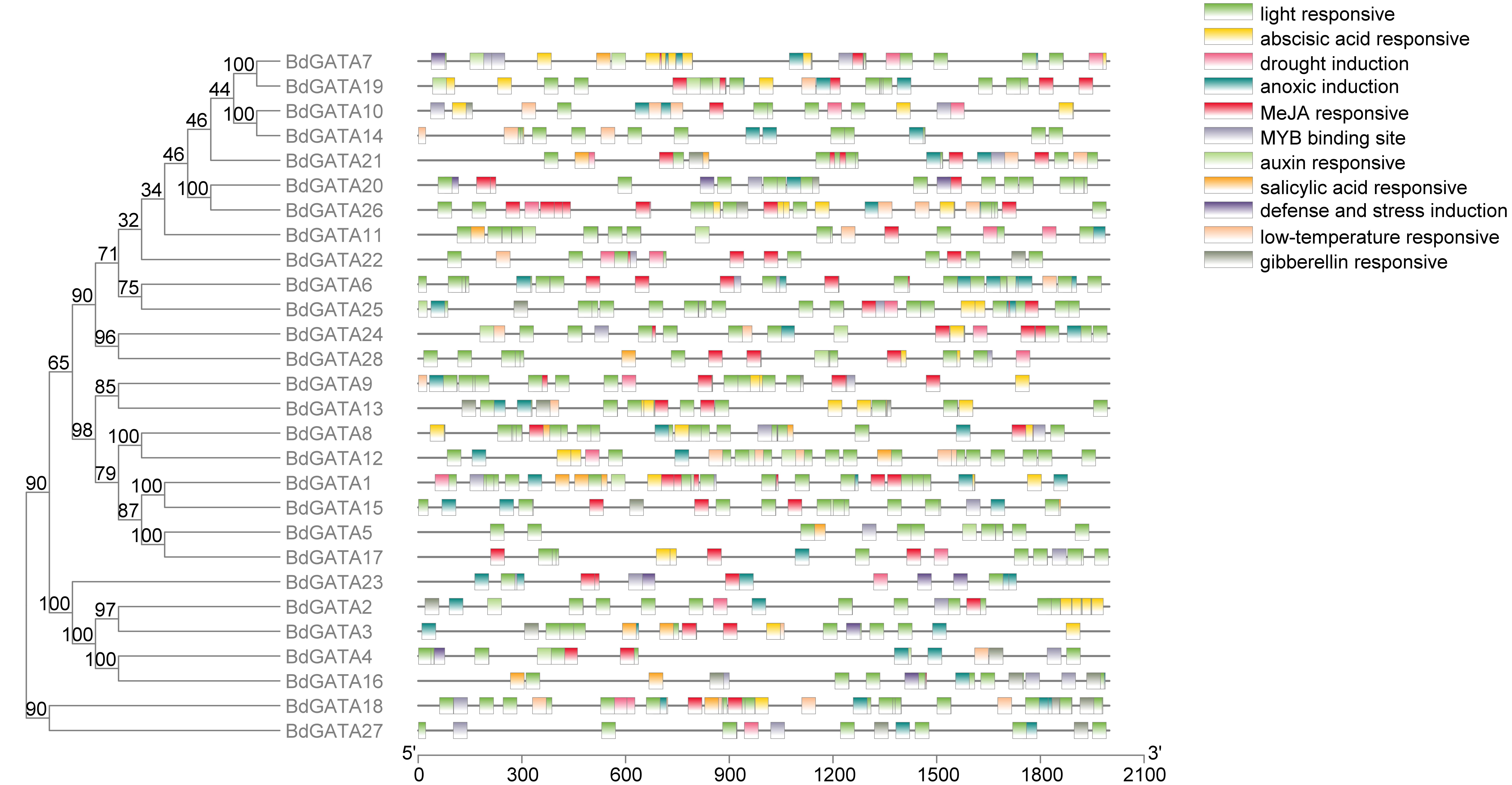

Supplement: Supplementary file 1 [file ijms-22-02026-s001.zip › supps resubmitted 2/Figure S2.jpg]
